# A Deep Analysis of the Small Non-Coding RNA Population in *Schistosoma japonicum* Eggs

**DOI:** 10.1371/journal.pone.0064003

**Published:** 2013-05-14

**Authors:** Pengfei Cai, Xianyu Piao, Lili Hao, Shuai Liu, Nan Hou, Heng Wang, Qijun Chen

**Affiliations:** 1 MOH Key Laboratory of Systems Biology of Pathogens, Institute of Pathogen Biology, Chinese Academy of Medical Sciences & Peking Union Medical College, Beijing, People's Republic of China; 2 Key Laboratory of Zoonosis, Ministry of Education, Institute of Zoonosis, Jilin University, Changchun, People's Republic of China; 3 Department of Microbiology and Parasitology, Institute of Basic Medical Sciences, Chinese Academy of Medical Sciences & School of Basic Medicine, Peking Union Medical College, Beijing, People's Republic of China; 4 College of Life Science and Technology, Southwest University for Nationalities, Chengdu, People's Republic of China; AC Camargo Cancer Hospital, Brazil

## Abstract

**Background:**

*Schistosoma japonicum* is a parasitic flatworm that causes zoonotic schistosomiasis. The typical outcome of schistosomiasis is hepatic granuloma and fibrosis, which is primarily induced by soluble egg-derived antigens. Although schistosomal eggs represent an important pathogenic stage to the host, the biology of this critical stage is largely unknown. We previously investigated the expression profiles of sncRNAs during different developmental stages of this parasite. However, using small RNA extracted from egg-deposited liver tissues generated limited information about sncRNAs in eggs. Here, we characterized the complete small RNAome in this stage of the parasite after optimization of RNA purification.

**Methodology and Principal Findings:**

A library, SjE, was constructed with the small RNA extracted from *S. japonicum* eggs and subjected to high-throughput sequencing. The data were depicted by comprehensive bioinformatic analysis to explore the expression features of sncRNAs in the egg stage. MicroRNAs accounted for about one quarter of the total small RNA population in this stage, with a strongly biased expression pattern of certain miRNA family members. Sja-miR-71, sja-miR-71-5p, and sja-miR-36-3p were suggested to play important roles in embryo development. A panel of transfer RNA fragments (tRFs) precisely processed from the 5′ end of mature tRNAs was identified for the first time, which represented a strong egg stage-biased expression. The tRNA-Ala derived small RNAs were the most highly expressed Sj-tRFs in eggs. Further, the expression of siRNAs from 29 types of well-defined transposable elements (TEs) was observed to be relatively stable among different developmental stages.

**Conclusions and Significance:**

In this study, we characterized the sncRNA profile in the egg stage of *S. japonicum*. Featured expression of sncRNAs, especially the tRNA-derived small RNAs, was identified, which was further compared with that of other developmental stages. These novel findings would facilitate a deeper understanding of the biology of schistosomal parasites.

## Introduction

Schistosomiasis, a debilitating disease, caused by blood flukes of the genus *Schistosoma* afflicts more than 230 million people worldwide (http://www.who.int/mediacentre/factsheets/fs115/en/index.html). The three major species infecting humans are *Schistosoma haematobium*, *S. mansoni*, and *S. japonicum*. The pathology of chronic infection with *S. japonicum* or *S. mansoni* is well known as hepatosplenic schistosomiasis, with clinical symptoms of granulomatous inflammation, periportal fibrosis, portal hypertension, hepatosplenomegaly, ascites, and the formation of vascular shunts [1], [2]. The granulomatous responses induced by schistosome soluble egg antigens (SEA) released from the eggshell-enclosed miracidium are regarded as an evolutionary compromise, that is critical for the survival of the infected host, and but also beneficial for the transmission of eggs [3]. As a classical immune regulatory model, the host immune responses induced by SEA were intensively investigated [4]–[7]; however, the gene expression regulatory mechanism during schistosomal embryonic development is still poorly understood.

Small non-coding RNAs (sncRNAs) with a size of 18∼30 nt have been found in most eukaryotes, and are increasingly recognized as powerful regulators of gene expression and genome stability [8], [9]. Among them, microRNAs (miRNAs), small interfering RNAs (siRNAs), and piwi-interacting RNAs (piRNAs) are the three major categories. So far, numerous miRNAs have been extensively identified in animals [10], plants [11], fungi [12], and viruses [13]. In mammals, mutation or deletion of enzymes involved in miRNA biogenesis has been observed to lead the defects in germ-line division and differentiation, and embryonic morphogenesis [14], [15]. In the nematode of *Meloidogyne incognita*, knockdown of drosha and pasha in undifferentiated eggs led to irregular growth and embryonic lethality [16]. Recent advances have also proved that non-coding RNAs (ncRNAs) are key regulators of embryogenesis, including miRNA-induced degradation of mRNAs and long ncRNA-mediated modification of chromatin [17]. Two other classes of sncRNAs, siRNAs and piRNAs, have known involvement in the defense against parasitic DNA elements to maintain genome stability. PiRNAs have been proposed to ensure germline stability in germ-line cells, whereas siRNAs were observed to play roles in both somatic and germ-line cells. These sncRNAs are loaded into the RNA-induced silencing complex (RISC) [18] or RNA-induced transcriptional silencing complex (RITS) [19] to function in chromatin architecture modelling, post-transcriptional repression and mRNA destabilization, mobile genetic elements suppression, and virus defence [8], [20]–[22]. In schistosome, several Argonaute orthologues were identified in both *S. japonicum* [23], [24] and *S. mansoni* [25], and one of the three Argonaute proteins in *S. japonicum*, SjAgo2 was suggested to maintain genome stability via suppression of retroelements [26].

In recent years, the knowledge regarding sncRNA biology has rapidly expanded within the phylum Platyhelminthes, using homology-based computational approach [27]–[29], molecular cloning methodology [27], [30]–[32], or deep sequencing techniques [33]–[38]. SncRNA profiles in other important parasitic nematodes have also been fractionally characterized [39]–[42]. We previously characterized the sncRNA profiles of *S. japonicum* at different developmental stages, including cercariae, lung-stage schistosomula, hepatic schistosomula, mixed and separated adult worms, and liver tissue-trapped eggs [36], [38]. However, using the RNA extracted from egg-deposited liver tissue for sequencing generates only limited information about sncRNAs in the egg stage of the parasite [38].

Since the tissue-trapped eggs are the major agents causing the severe pathology of schistosomiasis and those released from the host are relevant for the prevalence of the disease, it is indispensable to explore a complete repertoire of sncRNAs in schistosomal eggs, which will assist the discovery of novel intervention targets. In this study, small RNA extracted from purified *S. japonicum* eggs was subjected to high-throughput sequencing and deep analysis. The data provide a unique expression feature of egg sncRNAs, at a comparable level to those from other developmental stages of *S. japonicum*, which will shed light on the gene regulatory mechanisms during embryonic morphogenesis of the schistosomal parasite.

## Results and Discussion

### Isolation and purification of S. *japonicum* eggs

Rapid isolation of viable *S. japonicum* eggs from host hepatic tissue was a critical step for extracting intact total RNA. In our previous study [38], we analyzed the sncRNAs by directly sequencing the total small RNA from infected liver tissues. However, the host small RNA population, which overwhelmed that originating from the eggs, significantly reduced the resolution of the egg-derived small RNAome. In this study, an improved sieving and enzymatic methodology was applied. The purified egg samples were examined under a light microscope, and we found that most of the eggs contained a developing embryonic larva (miracidium) (Figure S1), whereas small-sized ones with immature embryos had either passed through the nylon mesh screens or were removed from the suspension in the Percoll column after centrifugation. Therefore, the data obtained here reflect mainly the sncRNA of mature *S. japonicum* eggs, which were able to release SEA to elicit the host hepatic granulomatous responses.

### General features of the two small RNA libraries of *S. japonicum* eggs

To investigate the small RNA profiles in the *S. japonicum* eggs, three libraries were constructed with small RNAs extracted from purified eggs and sequenced separately. Preliminary analysis indicated that one tRNA-derived small RNA fragment was preferentially amplified (≈32% of total reads) in one library, but not in the other two libraries. There were no significant differences in term of read numbers and sequence length distribution between the second and the third libraries (data not shown). Thus only the data of the second library were further analyzed. In total, 34,244,779 reads were generated by Solexa sequencing of the egg small RNA library (Table S1). In the library, ≈30.4 million reads were high-quality clean reads, which could be merged into 3,053,121 unique tags (Table S1 and S2). The redundancy of the library was 89.9 (Redundancy = 100−[Total Unique Clean Reads/Total high-quality Clean Reads×100]), which was higher than that of our previous small RNA libraries [36], [38]. The match rate of the library was more than 70% (Table S2), which is dramatically higher than that of our previous libraries SjE30 and SjE45 (both at ≈1%), which were constructed with total small RNA isolated from egg-trapped liver tissues [38]. Therefore, after eliminating contaminated reads derived from host tissue, the data presented here should reflect the authentic repertoire of sncRNA in *S. japonicum* eggs. Since the egg small RNA library has the same order of magnitude of reads number as other available *S. japonicum* small RNA libraries, it is possible to compare the profile and expression level of sncRNAs in the egg stage with that of other developmental stages.

### Length distribution of small RNA reads in different *S. japonicum* libraries

**Figure 1:**
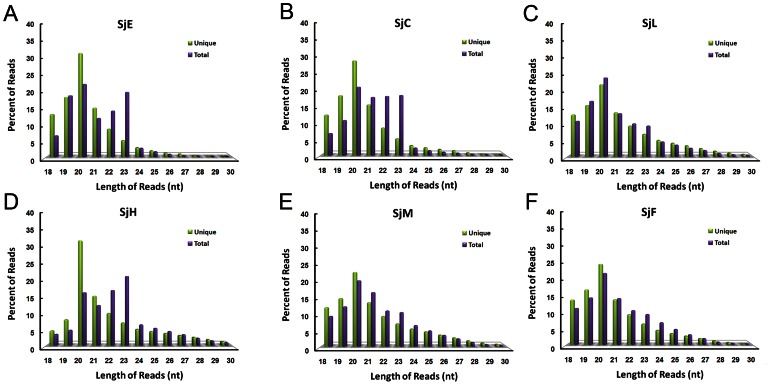
Length distribution of small RNA reads that perfectly matched the genome of *S. japonicum* in different libraries at unique and total levels. (A–F) Length distribution of the small RNA reads from small RNA libraries SjE (eggs), SjC (cercariae), SjL (lung-stage schistosomula), SjH (hepatic schistosomula), SjM (male adult worms), and SjF (female adult worms), respectively.

Here, we investigated the length distribution of small RNA reads from the egg small RNA library along with libraries of other developmental stages that perfectly matched the draft genome sequences of *S. japonicum*. As shown in Figure 1, the dominant species of small RNAs within *S. japonicum* are between 18 and 23 nt, which ruled out the piRNA (normally 29–31 nt) pathway in this species. The length distribution of the reads in SjE presented a pattern similar to that of SjC and SjH, at both total and unique levels, which featured as that the percentage of 22-nt and 23-nt reads, particularly the latter, was significantly higher at total level compare to that of unique level (Figure 1A, B, and D).

### Classification of sncRNAs in different small RNA libraries of S. *japonicum*

**Figure 2:**
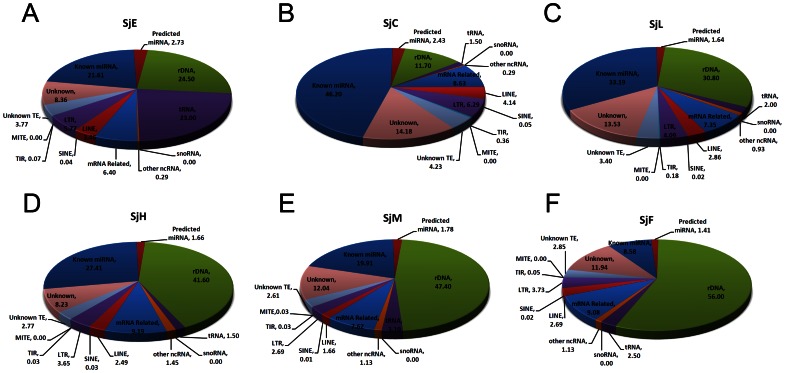
Classification and percentage of small non-coding RNAs in different small RNA libraries. (A–F) Classification of the small RNA reads from libraries SjE (eggs), SjC (cercariae), SjL (lung-stage schistosomula), SjH (hepatic schistosomula), SjM (male adult worms), and SjF (female adult worms), respectively. One significant feature was that the percentage of tRNA-derived small RNAs dramatically increased in the egg library,∼8–15 times more than that in other libraries. MiRNAs and rDNA-derived small RNA reads in the egg stage were at modest level compared to other stages.

The sncRNA transcripts in different small RNA libraries of *S. japonicum* were systematically analyzed using a more rigorous bioinformatic pipeline than previously described [38]. As shown in Figure 2, the percentage of miRNAs in the egg libraries accounted for ≈24%, which was slightly higher than that in the SjF library (9.65%), but lower than that in other three libraries (SjC, SjL, and SjH). The percentage of reads mapping to the 28S, 18S, and 5.8S rRNA genes, as well as the intergenic spacer sequences among these genes in the egg libraries was also modest (≈25%) when compared with that in the SjL, SjH, SjM, and SjF libraries. Unexpectedly, the reads derived from tRNAs were dramatically expanded in the egg libraries, compared with that of the other libraries. The tRNA-derived small RNA reads accounted for 23.0% of the RNA population in SjE, suggesting that there may be a specific processing mechanism of tRNA transcripts in the egg stage of the parasite. In addition, the TE-derived siRNAs in the egg libraries primarily originated from two types of retroelements, long terminal retrotransposons (LTRs) and long interspersed nucleotide elements (LINEs). There was no significant variation in the transcription of TE-derived siRNAs among different developmental stages of the parasite.

### MiRNAs expressed in S. *japonicum* eggs

**Figure 3:**
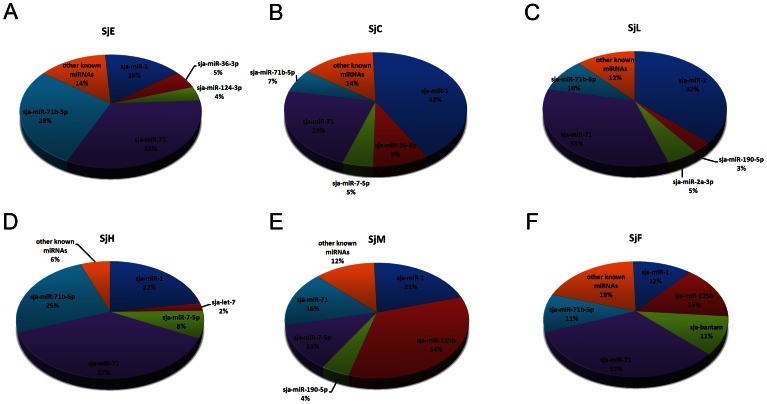
MiRNA family members with strongly biased expression at different developmental stages of *S. japonicum*. (A–F) The percentages of the top five expressed miRNAs along with the cohort of other less abundant known miRNAs were calculated based on TPM value in the small RNA libraries SjE (eggs), SjC (cercariae), SjL (lung-stage schistosomula), SjH (hepatic schistosomula), SjM (male adult worms), and SjF (female adult worms), respectively. The percentages of the top five miRNAs for each stage are shown separately, whereas the other less abundant miRNAs are bundled in the “other known miRNAs” category.

**Figure 4:**
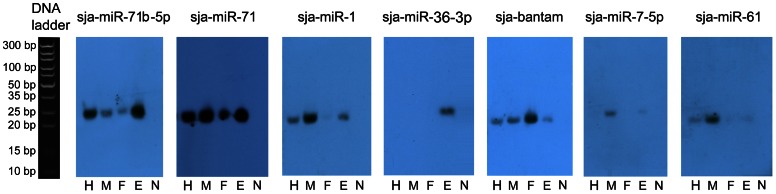
The expression of seven miRNAs at different developmental stages of *S. japonicum* was detected by Northern blot analysis. H, hepatic schistosomula; M, male adult worms; F, female adult worms; E, eggs; N, total RNA isolated from normal rabbit liver. M, Ultra low range DNA ladder denatured in RNA loading dye solution.

The clean reads from the six libraries were aligned to the 55 *S. japonicum* miRNA precursors in the Sanger miRBase [43], [44] (Release 18). In the egg libraries, 75 out of 78 known *S. japonicum* mature miRNAs were detected, significantly more than that of previous egg libraries (18 and 25 known mature miRNAs were found in the SjE30 and SjE45 libraries, respectively), suggesting that those miRNAs with low expression in eggs were detected in this study (Table S3). Of the miRNAs identified, sja-miR-71b-5p, sja-miR-71, sja-miR-1, sja-miR-36-3p, and sja-124-3p were the most abundant members at the egg stage (Figure 3A). These five miRNAs accounted for approximately 86% of all known miRNAs in the SjE library, which further supports our earlier finding that there is a strongly biased expression of particular miRNA families in each particular developmental stage of the parasite [38] (Figure 3). A similar phenomenon was also observed in other species, such as *Clonorchis sinesis*, in which members from the miR-71 family accounted for one third of the reads in the adult stage [37]. In the parasitic nematode *Trichinella spiralis*, members derived from the miR-1 and let-7 families were predominantly expressed in larvae [45]. Combining the TPM value of miRNAs (Table S3) and Northern blot analysis (Figure 4), we found that members of the sja-miR-71 family were the most highly expressed ones in the egg stage, implying that these miRNAs may play important regulatory functions during this stage.

The miR-36 family has so far been observed only in helminthes [46]. A conserved ortholog of sja-miR-36-3p was identified in *S. mansoni* by computational prediction [29]. Recently, Liu *et al*. also detected a putative miR-36 family member (tsp-Novel-21) in *T. spiralis*, which was mainly expressed in the adult worms [45]. The alignment of the Sj-miR-36-3 sequence with orthologs from other organisms is shown in Figure S2. All the orthologs shared a conserved seed sequence “CACCGGG” except bma-miR-36a and bma-miR-36b. In *C. elegans*, miR-36 was one of the eight functionally redundant members of the cel-miR-35 family (cel-miR-35∼42). Previously, Alvarez-Saavedra and colleagues comprehensively analyzed the function of the miR-35 family members in *C. elegans*, and found that mutation of seven members of this family led to developmental suppression, including embryonic and larval lethality [47]. More recently, this family was found to regulate the G1/S transition of intestinal cells and promote proliferation of germ cells in *C. elegans* [48]. Further, in the parasitic nematode of *Ascaris suum*, each asu-miR-36 family member was expressed in a narrow window from early to middle embryogenesis, implying that each member from this family may finely regulate the development of the parasite [41]. Considering the conserved seed sequence and its high expression in eggs compared to adult worms, it is postulated that sja-miR-36-3p may plays a similar role during embryonic development of *S. japonicum*. However, sja-miR-36-3p was also abundantly expressed in cercariae and lung-stage schistosomula (Table S3), suggesting that subtle regulatory mechanisms may be exerted at different developmental stages by one schistosomal miRNA.

### tRNA-derived small RNA in S. *japonicum*

**Figure 5:**
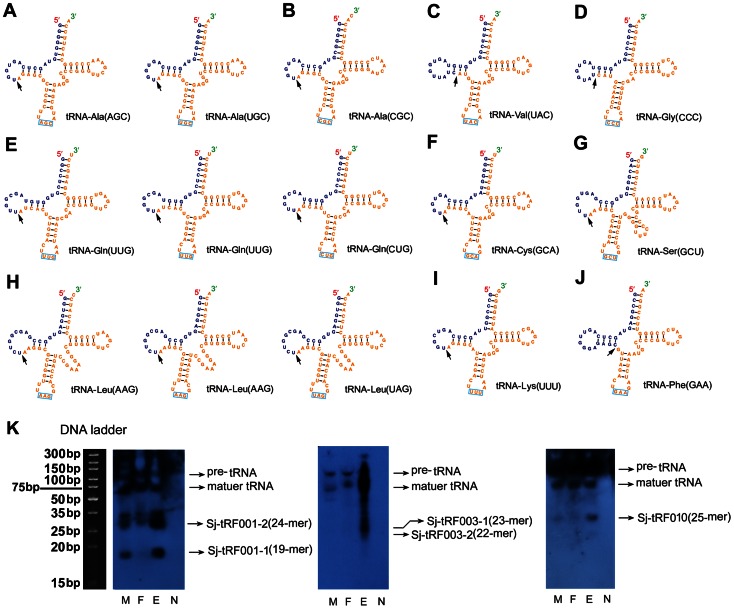
The schematic diagram of secondary structures of mature tRNAs and validation of Sj-tRFs expression in different stages of ***S. japonicum***. (A–J) The secondary structures of mature tRNAs, from which Sj-tRF001 ∼ Sj-tRF010 groups were identified, respectively. The 5′ end of tRNA transcripts for the generation of tRFs are represented in blue, the 3′ remnant in orange. Black arrows indicate the most frequent positions of splicing. Anticodons are boxed in light blue. (K) Northern blot analysis of the expression of the Sj-tRF001 (Sj-tRF002 and Sj-tRF006), Sj-tRF003, and Sj-tRF-010 groups in adult worms and eggs. Left panel, Sj-tRF001 group; middle panel, Sj-tRF003 group; right panel, Sj-tRF-010 group. M, male adult worms; F, female adult worms; E, eggs; N, total RNA isolated from normal rabbit liver. M, ultra low range DNA ladder denatured in RNA loading dye solution.

Previously, tRNA-derived RNA fragments (tRFs) precisely processed from mature or precursor tRNAs were detected in prostate cancer cell lines by ultra-high-throughput sequencing [49]. More recently, tRFs were identified in the halophilic archaeon *Haloferax volcanii* [50] and in the plant pathogenic fungus, *Magnaporthe oryzae* [51], indicating the existence of tRFs in various organisms. However, because the tRFs were found to be processed from different mature tRNAs by specific endonucleases (e.g., ELAC2 and Dicer) under stress responses and possibly on other occasions [52], tRFs may be suppressed at other developmental stages of *S. japonicum*, which may have prevented the identification of this group of small RNAs in our previous studies [36], [38]. Here, after mapping to the predicted *S. japonicum* tRNA sequences (sja.trna.bed), we unexpectedly found that small RNAs derived from tRNAs were abundantly present in the SjE library. The percentage of tRNA-derived small RNAs was significantly up-regulated in the egg stage compared with that of other stages (Figure 2). For the first time, we defined a panel of highly expressed Sj-tRFs processed from mature tRNAs of *S. japonicum* (Table 1). The secondary structures of the tRNAs for generating these Sj-tRFs are shown in Figure 5A–K.

**Table 1:** Sixteen Sj-tRFs identified in eggs and their relative expression levels in different small RNA libraries of *S. japonicum*.

Name	Sequence	Length	TPM						tRNA	Position
	(5′–3′)	(nt)	SjC	SjL	SjH	SjM	SjF	SjE		
Sj-tRF001-1	GGGGGUGUAGCUCAGUGGU	19	3982	624	113	1118	943	85833	tRNA-Ala(AGC)/(UGC)	5′
Sj-tRF001-2	GGGGGUGUAGCUCAGUGGUAGAGC	24	124	23	503	66	22	124	tRNA-Ala(AGC)/(UGC)	5′
Sj-tRF001-3	GGGGGUGUAGCUCAGUGGUA	20	1313	2710	478	5257	4285	1294	tRNA-Ala(AGC)/(UGC)	5′
Sj-tRF001-4	GGGGGUGUAGCUCAGUGG	18	141	37	15	90	105	620	tRNA-Ala(AGC)/(UGC)	5′
Sj-tRF002	GGGGGCGUAGCUCAGUGGU	19	31	246	5	145	74	550	tRNA-Ala(CGC)	5′
Sj-tRF003-1	GGUUCGGUGGUGUAGUGGUUAUC	23	101	144	265	214	171	20281	tRNA-Val(UAC)	5′
Sj-tRF003-2	GGUUCGGUGGUGUAGUGGUUAU	22	204	140	436	290	228	20085	tRNA-Val(UAC)	5′
Sj-tRF004-1	GCGCCGGUAGUGUAGCGGUAU	21	56	82	59	153	439	14508	tRNA-Gly(GGG)	5′
Sj-tRF004-2	GCGCCGGUAGUGUAGCGGUAUC	22	34	275	73	294	432	5539	tRNA-Gly(GGG)	5′
Sj-tRF005	GGCCUCGUGGUGUAGCGGUU	20	135	242	264	643	567	12524	tRNA-Gln(UUG)/(CUG)	5′
Sj-tRF006	GGGGGUAUAGCUCAGUGGU	19	2	3	2	4	3	130	tRNA-Cys(GCA)	5′
Sj-tRF007	GACGGGGUGGCCGAGUGGUU	20	15	14	3	6	19	2410	tRNA-Ser(GCU)	5′
Sj-tRF008-1	GGUGGAGUGGCCGAGCGGUCU	21	50	44	308	50	79	2486	tRNA-Leu(AAG)/(UAG)	5′
Sj-tRF008-2	GGUGGAGUGGCCGAGCGGU	19	129	14	118	5	28	2420	tRNA-Leu(AAG)/(UAG)	5′
Sj-tRF009	GCCCGGUUAGCUCAGUCGGU	20	59	31	17	145	63	2284	tRNA-Lys(UUU)	5′
Sj-tRF010	GCCGGAGUAGCUCAGUUGGGAGAGC	25	21	71	209	88	63	61	tRNA-Phe(GAA)	5′

Previous studies indicated that tRFs can be derived from different positions within tRNAs [49]. Lee *et al*. categorized tRFs into three series, of which tRF-5 and tRF-3 were aligned to the 5′ and 3′ ends of mature tRNA, respectively, whereas the tRF-1 series are located within pre-tRNA, and their 5′ ends start precisely after the 3′ ends of the mature tRNA sequence [49]. Here, we found that the tRFs in *S. japonicum* were preferentially processed from the 5′ end of mature tRNAs (Table 1 and Figure 5A–K), which represent an extensive terminal and asymmetric processing of tRNA, as recently reported [53]. Furthermore, like that observed in miRNAs, isoforms from particular tRNA(s), such as Sj-tRF-001–1∼Sj-tRF-001–4, were also commonly identified (Table 1).

The tRF, Sj-tRF-001–1, which could be processed from mature tRNA-Ala (AGC) and tRNA-Ala (UGC) (Figure 5A), represented the most abundant read deposited in the SjE library. However, the hybridization signal corresponding to 24-nt small RNAs was stronger than that of 19-nt (Figure 5J, left panel), which could be due to the cross-hybridization to some other tRFs homologous to Sj-tRF-001–2. In addition, Sj-tRF-002 and Sj-tRF-006, which are respectively derived from tRNA-Ala (CGC) and tRNA-Cys (GCA), are highly homologous to Sj-tRF-001–1, with only one base mismatch. Therefore, the signal at the position of 19-nt may reflect the expression of all three tRFs (Figure 5J, left panel). Sj-tRF-003–1 and Sj-tRF-003–2 exhibited a biased expression in eggs as well as their pre- and mature tRNAs (Figure 5J, middle panel). Moreover, a relatively low expressed tRF, Sj-tRF-010, was also detected mainly in the egg stage (Figure 5J, right panel). Although the biogenesis and function of tRFs remains to be further clarified, one exciting finding reported by Lee *et al*, was that tRF-1001 can regulate cell proliferation in prostate cancer cell lines [49]. As tRFs are predominantly expressed in the egg stage of *S. japonicum*, we postulate that some of these tRFs may play a role in regulating the development of schistosomal embryos.

### Small RNAs mapping to *S. japonicum* rDNA repeats

**Figure 6:**
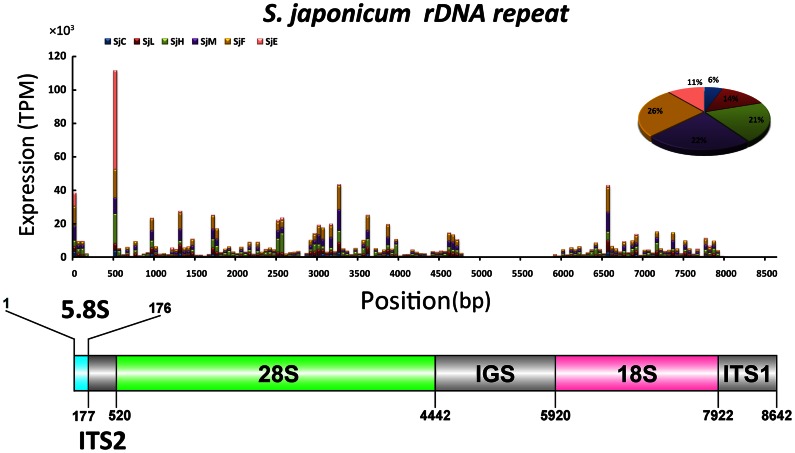
The distribution and abundance of sense small RNAs in the six small RNA libraries matched the rDNA repeat sequence of *S. japonicum*. The percentage of total small RNAs mapping to the rDNA repeat sequence for each library was calculated by using the sum of TPM value of all small RNA reads and was displayed in the pie charts. The gene structure of rDNA repeat was indicated in the lower panel and the positions of gene elements were corresponded to the upper panel. The expression of antisense small RNAs matched to these elements were considerably low, and they were not shown in the image. ITS, internal transcribed spacer; IGS, intergenic spacer region.

Deep analysis of the small RNA identified a large proportion of sncRNAs that were derived from rDNA, especially in the hepatic schistosomula and adult worms (Figure 2). A similar result was also observed by Xue *et al.*, who observed an over-representation of fragmented rRNAs in the pool of short RNAs in their *S. japonicum* library [32]. They suggested that processing of the 28S large rRNA subunit (the phenomenon known as “nick in vivo” in flatworms) may be the source of the large proportion of rRNA fragments. After precisely mapping the small RNAs to the ribosomal DNA repeat sequences (5.8S-ITS2-28S-IGS-18S-ITS1) of *S. japonicum*, we confirm that the majority of these small RNAs were derived from the 28S rRNA (Figure 6). Notably, the amount of small RNA reads, 5′-CUGACCUCGGAUCAGACGUGAU(U)-3′, derived from the 5′-terminus of 28S rRNA was predominant in the SjE library. Since the highly integrity of the total RNA extracted from the eggs (Figure S3), it is suggested that the high-abundant rRNA fragments derived from specific regions of the 28S rRNA were not due to the random degradation of rRNA transcripts. Whether these small RNAs are functionally processed products of rRNA, as in the case of tRNA-derived sncRNAs, remains to be determined.

### Expression of 29 well-defined TE-derived endo-siRNAs in S. *japonicum*

**Figure 7:**
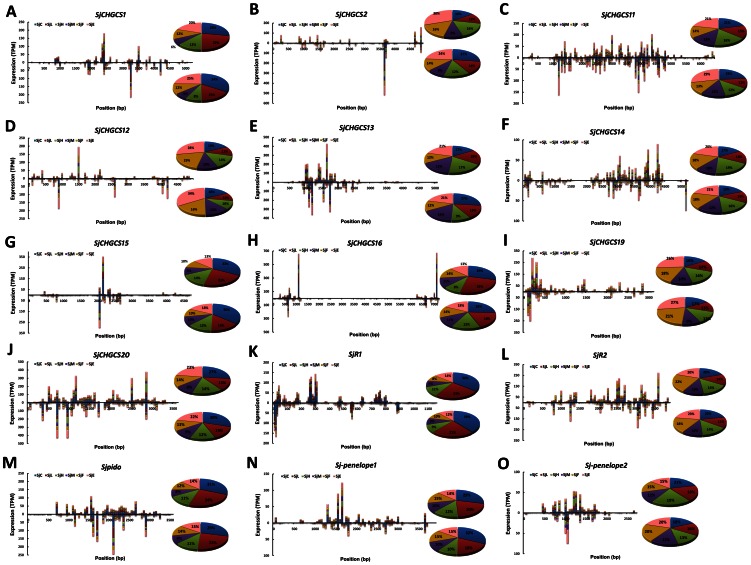
The distribution and abundance of TE-derived siRNAs in different small RNA libraries of *S. japonicum*. The expression of sense and antisense siRNA reads generated from one particular TE was normalized as TPM value, and bars with different colors were created to indicate the abundance of retrotransposon-derived siRNAs in different libraries. (A–H) Endo-siRNAs derived from the LTR retrotransposons *SjCHGCS1*, *SjCHGCS2*, *SjCHGCS11*, *SjCHGCS12*, *SjCHGCS13*, *SjCHGCS14*, *SjCHGCS15*, and *SjCHGCS16*, respectively. (I–M) Endo-siRNAs derived from non-LTR retrotransposons, *SjCHGCS19*, *SjCHGCS20*, *SjR1*, *SjR2*, and *Sjpido*, respectively. (N) Endo-siRNAs mapping to the Penelope-like element retrotransposons *Sj-penelope-1* and (O) *Sj-penelope-2*. The percentage of total siRNAs derived from one particular TE for each library was calculated by using the sum of TPM value of each siRNA and was displayed in the pie charts. The upper panel in each chart represents sense siRNAs; the lower panel in each chart represents antisense siRNAs.

TE sequences constitute 21.84% of the *S. japonicum* genome [54], and the mobile genetic elements (MGEs) in the *S. japonicum* genome, including short interspersed nucleotide elements (SINEs)-like retrotransposons, LTR retrotransposons, non-LTR retrotransposons, and *penelope*-like retrotransposons, have been characterized in several studies [54]–[56]. Some of these elements are still actively mobile within the genome [55], implying that they must be under tight regulation to maintain the stability of the genome [57]. We recently demonstrated that TE-derived siRNAs in *S. japonicum* were at least partially associated with the Argonaute protein, Ago2 [26]. In this study, the reads from the six libraries were mapped to the 29 well-defined TEs, including 18 LTR retrotransposons (*SjCHGCS1-18*), 7 non-LTR retrotransposons (*SjCHGCS19-22*, *SjR1*, *SjR2*, and *Sjpido*), 3 *Penelope*-like retrotransposons (*Sj-penelope1-3*), and *Gulliver*. The expression levels of siRNAs derived from these retrotransposons in the related libraries were presented based on their TPM value (Table 2). We found that siRNAs derived from these 29 well-defined TEs were stably expressed at different developmental stages, though slightly suppressed in the male adult worms (Figure 7). However, the expression of siRNAs derived from different TEs was relatively diverse; for instance, the siRNAs originating from *Gulliver*, *SjCHGCS18*, *SjCHGCS21*, *SjCHGCS2*2, and *Sj-penelope3* were present nearly at undetectable levels, whereas those from *SjCHGCS3* were highly expressed. In addition, the expression of both sense and antisense siRNAs from one specific TE showed a symmetrical pattern, with the exception of *SjCHGCS17*, from which the antisense siRNAs are predominant (Table 2). No major variation in expression level was observed in all libraries, supporting the earlier speculation that the main function of TE-derived siRNAs was to maintain the genomic stability of *S. japonicum* [26].

**Table 2:** Expression levels of siRNAs derived from 29 well-defined retrotransposons in different small RNA libraries.

TE types	GenBankAccessionNo.	Sense (TPM)						Antisense (TPM)					
		SjC	SjL	SjH	SjM	SjF	SjE	SjC	SjL	SjH	SjM	SjF	SjE
*SjR1*	AF073333	313.3	199.2	92.5	69.8	74.9	123.3	249.5	136.0	57.2	54.2	60.6	74.2
*SjR2*	AY027869	1568.5	1120.9	1116.7	829.9	1741.0	1567.5	1317.0	805.1	814.5	612.9	1106.2	1361.5
*Sjpido*	AY034003	963.1	725.4	351.1	235.7	363.7	424.1	1049.0	801.2	401.4	319.2	483.9	542.2
*Gulliver*	AF243513	11.2	9.0	1.2	2.7	7.1	4.7	2.1	2.7	0.8	0.8	1.3	2.3
*SjCHGCS1*	FN356203	731.9	572.1	306.1	157.0	314.8	516.7	948.3	425.5	211.3	169.8	329.4	675.9
*SjCHGCS2*	FN356204	431.9	356.3	459.1	245.1	432.7	838.9	964.0	502.6	438.0	297.5	474.5	851.3
*SjCHGCS3*	FN356205	4842.1	2487.4	2033.4	1571.3	2680.5	4508.1	1550.3	947.4	914.4	591.4	1406.4	1356.0
*SjCHGCS4*	FN356206	358.9	152.8	104.3	106.3	136.1	202.1	221.8	241.0	163.3	125.7	216.3	159.2
*SjCHGCS5*	FN356207	117.4	49.6	50.5	27.8	43.0	57.2	128.3	60.8	48.4	30.8	43.2	66.7
*SjCHGCS6*	FN356208	282.4	238.6	378.1	237.2	268.7	608.5	358.9	191.4	248.2	251.5	290.7	655.2
*SjCHGCS7*	FN356209	973.0	775.8	311.8	400.2	810.4	696.9	340.2	319.3	237.5	169.2	279.0	241.1
*SjCHGCS8*	FN356210	107.3	101.4	43.3	48.8	120.6	88.8	16.6	15.8	15.6	9.1	24.9	17.8
*SjCHGCS9*	FN356211	135.0	160.2	122.4	127.1	152.7	128.8	154.6	127.7	89.4	64.3	94.7	105.8
*SjCHGCS10*	FN356212	704.3	250.1	364.0	108.8	226.9	322.1	548.6	152.8	137.1	98.1	164.4	406.2
*SjCHGCS11*	FN356213	1020.6	739.2	759.7	618.0	665.0	1048.5	1518.3	738.8	779.0	746.8	815.9	1843.3
*SjCHGCS12*	FN356214	396.7	253.6	310.7	219.1	410.9	614.2	539.6	304.0	294.2	351.8	493.5	1021.3
*SjCHGCS13*	FN356215	940.1	1033.8	989.8	876.4	705.1	1180.7	1596.9	867.2	538.3	942.8	727.8	1244.3
*SjCHGCS14*	FN356216	380.7	360.5	419.8	224.5	418.8	464.3	370.7	272.5	306.9	236.9	356.7	413.7
*SjCHGCS15*	FN356217	702.9	449.2	290.0	190.0	206.8	289.9	930.3	365.0	294.5	251.8	259.5	455.5
*SjCHGCS16*	FN356218	1725.0	1450.0	499.2	635.0	819.2	771.4	831.2	574.9	430.8	339.7	442.3	564.5
*SjCHGCS17*	FN356219	213.6	191.0	51.3	93.4	85.9	68.7	2725.6	1945.2	1107.7	1047.8	1044.4	983.9
*SjCHGCS18*	FN356220	1.6	0.8	0.0	0.4	0.4	3.3	0.4	0.4	1.2	0.0	0.1	1.2
*SjCHGCS19*	FN356221	821.0	709.3	779.7	510.0	885.9	1293.1	479.0	363.2	355.7	245.5	600.8	760.5
*SjCHGCS20*	FN356222	2741.5	1335.0	1461.4	923.6	1369.1	2285.4	2853.7	1315.0	1116.9	895.0	1204.2	2069.2
*SjCHGCS21*	FN356223	98.7	71.3	52.5	97.7	24.3	80.4	201.4	148.8	126.3	221.6	59.9	146.5
*SjCHGCS22*	FN356224	12.7	1.7	3.2	1.3	3.6	3.9	1.3	1.0	0.0	0.4	0.3	0.2
*Sj-penelope1*	FN356225	488.6	339.0	207.6	174.8	249.4	227.5	340.7	190.0	99.3	103.9	161.5	158.4
*Sj-penelope2*	FN356226	244.8	210.3	219.3	130.4	171.0	168.4	181.1	157.5	126.3	132.1	201.3	202.3
*Sj-penelope3*	FN356227	0.3	0.8	0	0.6	0.9	0.3	6.7	2.4	1.6	4.2	7.1	3.2

In summary, we have further dissected the expression characteristics of the small RNAome in the egg stage of *S. japonicum*. Strong biased expression patterns of certain miRNA family members were observed, of which, the expression of sja-miR-71, sja-miR-71-5p, and sja-miR-36-3p were prominent in this pathologically-related stage. Transfer RNA (tRNA)-derived small RNA fragments, precisely processed mainly from the 5′ side of tRNA transcripts, were identified for the first time as a novel class of small RNA in *S. japonicum*, which exhibited a significant stage-biased expression pattern, indicating their potential regulatory function in this stage. The most highly expressed tRF, Sj-tRF-001-1, has the potential to serve as an egg stage-specific bio-marker. The TE-derived siRNAs, which showed less variation in expression among different stages, also appeared to be an important constituent of the small RNA population, and is likely to protect the integrity of the genome against retroelements. The data in this study provide novel insights into the small RNAome of *S. japonicum*, which will facilitate a deeper understanding of the biology of this important parasitic pathogen.

## Materials and Methods

### Animals and Parasites

*S. japonicum*-infected *Oncomelania hupensis* snails were purchased from Jiangxi Provincial Institute of Parasitic Diseases, Nanchang, China. The cercariae were shed by exposing the snails to light conditions. A total of six New Zealand white rabbits were randomly assigned to two groups. Each rabbit was percutaneously infected with∼1,200 cercariae. Hepatic schistosomula and mixed sex adult worms were recovered at 2 and 6 weeks post-infection, respectively, by hepatic-portal perfusion from the infected rabbits. Male and female worms were manually separated with the aid of a light microscope, and washed three times with phosphate buffered saline (PBS). Liver tissues were also obtained from the infected rabbits at 6 weeks post-infection. All procedures carried out on animals within this study were conducted following the animal husbandry guidelines of the Chinese Academy of Medical Sciences and with permission from the Experimental Animal Committee of the Chinese Academy of Medical Sciences with the Ethical Clearance Number IPB-2011-6.

### Egg isolation

The schistosomal eggs were isolated by an improved sieving and enzymatic method [58]. The egg-trapped liver tissues were chopped with a scalpel blade and homogenated to a smooth consistency in 500 ml ice-cold PBS. The suspension was successively passed through 80, 120, 160, 200, and 260 mesh metal sieves, and finally a 320 mesh nylon screen. After repeated washes with PBS, the eggs on the nylon screen were collected in a 50 ml tube. The eggs were washed three times by discarding the tissue debris-containing suspension after natural sedimentation on ice. The pellet was resuspended in 50 ml PBS containing 10 mg collagenase B, 125 mg trypsin, 10 µg penicillin, and 20 µg streptomycin, then incubated at 37°C for 3 h with gentle shaking. The sample was then centrifuged at 1,500 rpm at 4°C for 5 min, and the supernatant was removed. This washing procedure was repeated twice more. The egg pellet was resuspended in 2 ml PBS and layered on the top of a Percoll column (containing a mixture of 2.4 ml of Percoll and 9.6 ml of 0.25 M sucrose) in a 15 ml tube. The tube was centrifuged at 2,000 rpm at 4°C for 5 min. Liver debris remaining in the supernatant was removed. The eggs were resuspended in 2 ml PBS for two more Percoll separations. The egg pellet was washed for 3 times with PBS then transferred to 1.5 ml tubes. The purity and integrity of the eggs was examined with the aid of a light microscope.

### Total RNA preparation

After centrifuging at 12,000 rpm for 1 min, the egg pellet was ground in liquid nitrogen. Total RNA from the eggs was extracted using Trizol reagent (Invitrogen, CA, USA) according to the manufacturer's protocol. Total RNA from hepatic schistosomula, male adult worms, female adult worms, and normal rabbit livers were also isolated using Trizol reagent. RNA quantification and quality were examined with a Nanodrop ND-1000 spectrophotometer (Nanodrop Technologies, Wilmington, DE) and standard agarose gel electrophoresis. All RNA samples were stored at −80°C until use.

### Small RNA library construction and deep sequencing

The egg total RNA sample was evaluated with an Agilent 2100 Bioanalyzer before library construction (Figure S3). Three small RNA libraries were constructed as described previously [36]. Small RNAs between 15–30 nucleotides (nt) were recovered from a 15% TBE-Urea polyacrylamide gel electrophoresis (PAGE), and ligated into Illumina's proprietary 5′ and 3′ adaptors. The product was converted into single-stranded cDNA using Superscript III reverse transcriptase (Invitrogen, CA, USA). The cDNA was then amplified with Illumina's small RNA primer pair using Phusion high-fidelity DNA polymerase (NEB) in 18 PCR cycles. The purified PCR products were sequenced using Illumina's Genome Analyzer platform at the BGI (Beijing Genomics Institute, Shenzhen, China).

### Bioinformatics analysis of reads from different small RNAs libraries of S. *japonicum*

Raw datasets were produced by deep sequencing of three libraries. After primary analysis, only the data generated from one library, designated as SjE, were further analyzed. The data were simultaneously analyzed with the previous small RNA datasets from cercariae, lung-stage schistosomula, hepatic schistosomula, male adult worms and female adult worms. First, the low quality reads, adaptor null reads, insert null reads, 5′ adaptor contaminants, and reads with poly(A) tail were filtered. Adapter sequences were then trimmed from both ends of clean reads. Clean reads were obtained after all identical sequences were counted and merged as unique sequences These unique sequences were mapped onto the *S. japonicum* genome of SGST (http://lifecenter.sgst.cn) using the program SOAP version 2.20 [59]. We investigated the length distribution of the perfectly matched small RNA reads in the six libraries [38]. Further, these small RNA reads were categorized using an optimal bioinformatic pipeline. In our previous study [38], analysis was focused mainly on miRNAs, whereas here, the unique sequences originating from snoRNAs (small nucleolar RNA) were in the first step filtered out [60], and sequences of rRNAs and tRNAs were investigated separately. In detail, 28S, 18S, and 5.8S rRNA sequences and rRNA intergenic spacer sequences (GenBank Accession Number: Z46504.4 [48], AY157226.1 [61], FJ852569.1 [62], and EU835685.1 [63]) of *S. japonicum* were retrieved from the NCBI GenBank database [64]. The putative tRNA gene sequences of *S. japonicum* were downloaded from http://www.bioinf.uni-leipzig.de/publications/supplements/08-014 [28]. Reads from different small RNA libraries mapped to those rRNA and tRNA sequences, other than to the data of Rfam as in the previous study [38], were respectively defined as rRNA-derived and tRNA-derived small RNAs. The secondary structures of mature tRNAs were predicted using an on line algorithm, ARAGORN [65]. The remaining perfectly matched reads were then BLAST-searched against the 77 known mature miRNAs of *S. japonicum* deposited in the Sanger miRBase [43], [44] (Release 18) using the program Patscan [66], and were further BLAST-searched against the conserved and novel *S. japonicum* miRNAs reported in our previous study [38]. Next, the reads were matched to the transposable elements in the *S. japonicum* genome predicted by using REPET software (http://urgi.versailles.inra.fr/index.php/urgi/Tools/REPET), in the order of LINE (Long Interspersed Elements), SINE (Short Interspersed Elements), LTR (Transposable elements with Long Terminal Repeats), TIR (Terminal inverted repeat), MITE (Miniature inverted-repeat transposable elements), and unknown TEs. The remaining small RNAs were aligned to *S. japonicum* predicted mRNA sequences (sjr_mRNA.fasta) downloaded from SDSPB using SOAP 2.20 aligner, and perfectly matched reads were retained as mRNA-related siRNA. Finally, the remaining reads were labeled as unknown small RNAs. We employed IDEG6 [67] to identify miRNAs or tRFs showing statistically significant difference in relative abundance (as reflected by TPM, transcripts per million) between any two small RNA libraries. The general Chi-square test was applied to determine whether one particular miRNA or tRF was significantly differentially expressed between any two samples (*P* value< = 0.01) (Table S3 and S4).

To further characterize the small RNAome, full length sequences of 29 classes of retrotransposons [54], [56], [68], [69] were retrieved from the NCBI GenBank database [64]. The small RNA reads from the libraries SjC, SL, SjH, SjF, SjM, and SjE were mapped to the sequences of rDNA repeat and the above mentioned retrotransposons. The abundance of rDNA-derived small RNAs or retrotransposon-derived siRNAs was reflected based on their expression values (TPM). A set of graphs depicting the distribution and abundance of these small RNAs was constructed as previously described [26]. All sequence data of the six small RNA libraries have been submitted to NIH Short Read Archive with the Accession numbers of SRR786675 (for SjE), SRR786666 (for SjC), SRR786671 (for SjL), SRR786672 (for SjH), SRR786673 (for SjM), and SRR786674 (for SjF).

### Confirmation of sncRNA expression by Northern blot

The 5′-DIG-labeled miRCURY LNA probes were synthesized by Exiqon (Vedbaek, Denmark) (Http://www.exiqon.com) (Table S5). Northern blot analysis was performed as described previously [70]. Total RNAs (10 µg each) from different *S. japonicum* stages were resolved by 15% denaturing PAGE (7 M urea). The samples were then transferred to neutral nylon membranes (Hybond-NX, GE) by capillary with 20×SSC, and cross-linked to the membrane using an EDC (1-ethyl-3-(3-dimethylaminopropyl) carbodiimide) method [71]. The blots were rinsed thoroughly with double distilled water and pre-hybridized at 37°C for 3 h in DIG Easy Granule (Roche). Hybridization was carried out in fresh DIG Easy Granule containing 1 nM DIG-labeled LNA probe at the recommended temperature (RNA Tm−30°C) overnight. Blots were then washed sequentially in a low stringency buffer (2×SSC, 0.1% w/v SDS) and a high stringency buffer (0.1×SSC, 0.1% w/v SDS) at the hybridization temperature. After briefly rinsing in washing buffer, the blots were incubated in blocking buffer at room temperature for at least 2 h (DIG washing and blocking buffer Set, Roche). Subsequently, the blots were incubated with a 10,000-fold dilution of anti-DIG-AP Fab fragment (Roche) in blocking buffer at room temperature for 30 min then washed 5 times for 15 min each in washing buffer. Blots were then rinsed in detection buffer for 5 min. Anti-DIG-AP was detected using CDP-star chemiluminescent substrate for alkaline phosphatase (Roche). Blots were stripped by boiling for 1 min at 100°C in 10 mM Tris-HCl, 5 mM EDTA, and 0.1% SDS and probed up to three times.

## Supporting Information

Figure S1 **Viable** ***S. japonicum*** **eggs purified from the hepatic tissues of infected rabbits.** A majority of the eggs contains a developing miracidium.
(JPG)

Figure S2 **Sequence alignment of sja-miR-36-3p with its orthologs from other species.** Alignment of sja-miR-36-3p with homologous sequences from *S. mansoni* (sma), *S. mediterranea* (sme), *A. suum* (asu), *Capitella teleta* (cte), *T. spiralis* (tsp), *Caenorhabditis briggsae* (cbr), *C. elegans* (cel), *Brugia malayi* (bma), *D*. *melanogaster* (dme), *Drosophila mojavensis* (dmo), *Apis mellifera* (ame), *Bombyx mori* (bmo), *Tribolium castaneum* (tca) and *Anopheles gambiae* (aga), was performed by DNAMAN version 6.0 and further refined with GeneDoc software.
(TIF)

Figure S3 **Agilent 2100 Bioanalyzer analysis of total RNA sample extracted from the purified eggs.**
(TIF)

Table S1 **General information of the** ***S. japonicum*** **egg small RNA library, SjE.**
(XLS)

Table S2 **Data statistics of the** ***S. japonicum*** **egg small RNA library, SjE.**
(XLS)

Table S3 **Detailed information of all known miRNAs expressed in different small RNA libraries of** ***S. japonicum*** **.**
(XLS)

Table S4 **Statistical analysis for determining whether one specific tRF was significantly differentially expressed between any two small RNA libraries.**
(XLS)

Table S5 **The information of the probes used in Northern blot analysis.**
(XLS)
