# Supplementary figures and images for: A Deep Analysis of the Small Non-Coding RNA Population in Schistosoma japonicum Eggs
Source: PLoS One. 2013 May 14;8(5):e64003. doi: 10.1371/journal.pone.0064003 (PMC3653858; doi:10.1371/journal.pone.0064003)

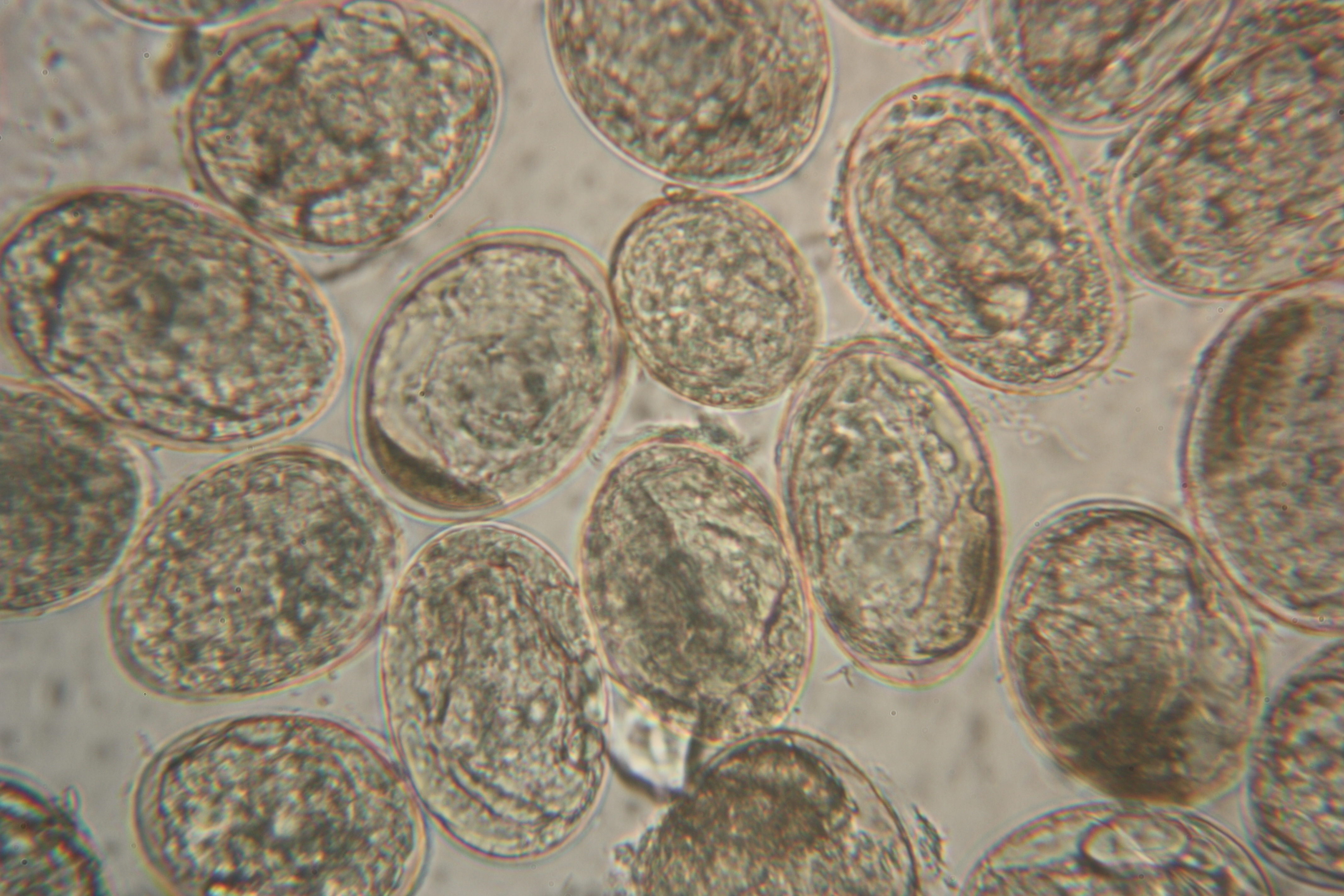

Supplement: Figure S1 — Viable S. japonicum eggs purified from the hepatic tissues of infected rabbits. A majority of the eggs contains a developing miracidium. (JPG) [file pone.0064003.s001.jpg]

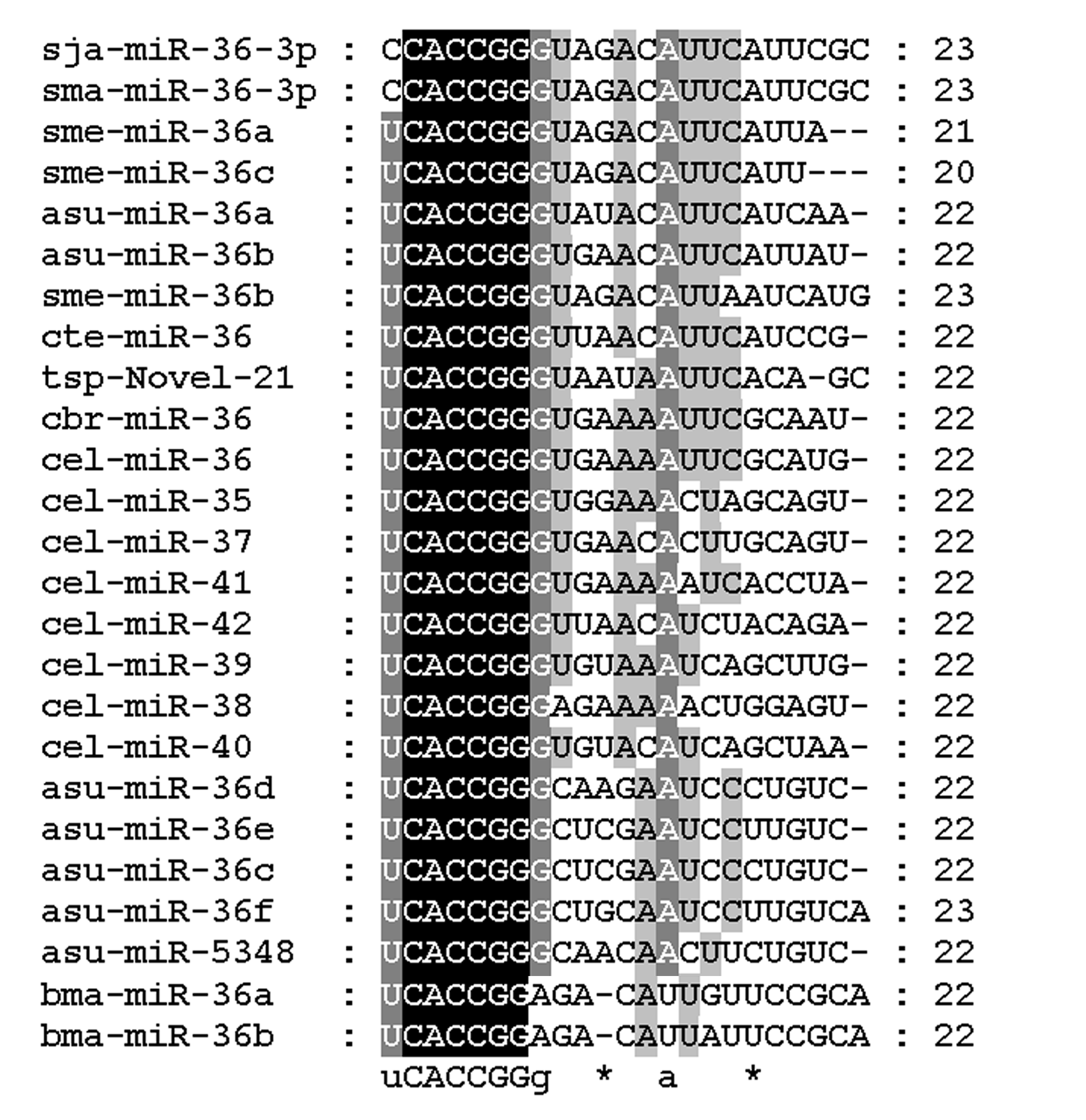

Supplement: Figure S2 — Sequence alignment of sja-miR-36-3p with its orthologs from other species. Alignment of sja-miR-36-3p with homologous sequences from S. mansoni (sma), S. mediterranea (sme), A. suum (asu), Capitella teleta (cte), T. spiralis (tsp), Caenorhabditis briggsae (cbr), C. elegans (cel), Brugia malayi (bma), D. melanogaster (dme), Drosophila mojavensis (dmo), Apis mellifera (ame), Bombyx mori (bmo), Tribolium castaneum (tca) and Anopheles gambiae (aga), was performed by DNAMAN version 6.0 and further refined with GeneDoc software. (TIF) [file pone.0064003.s002.tif]

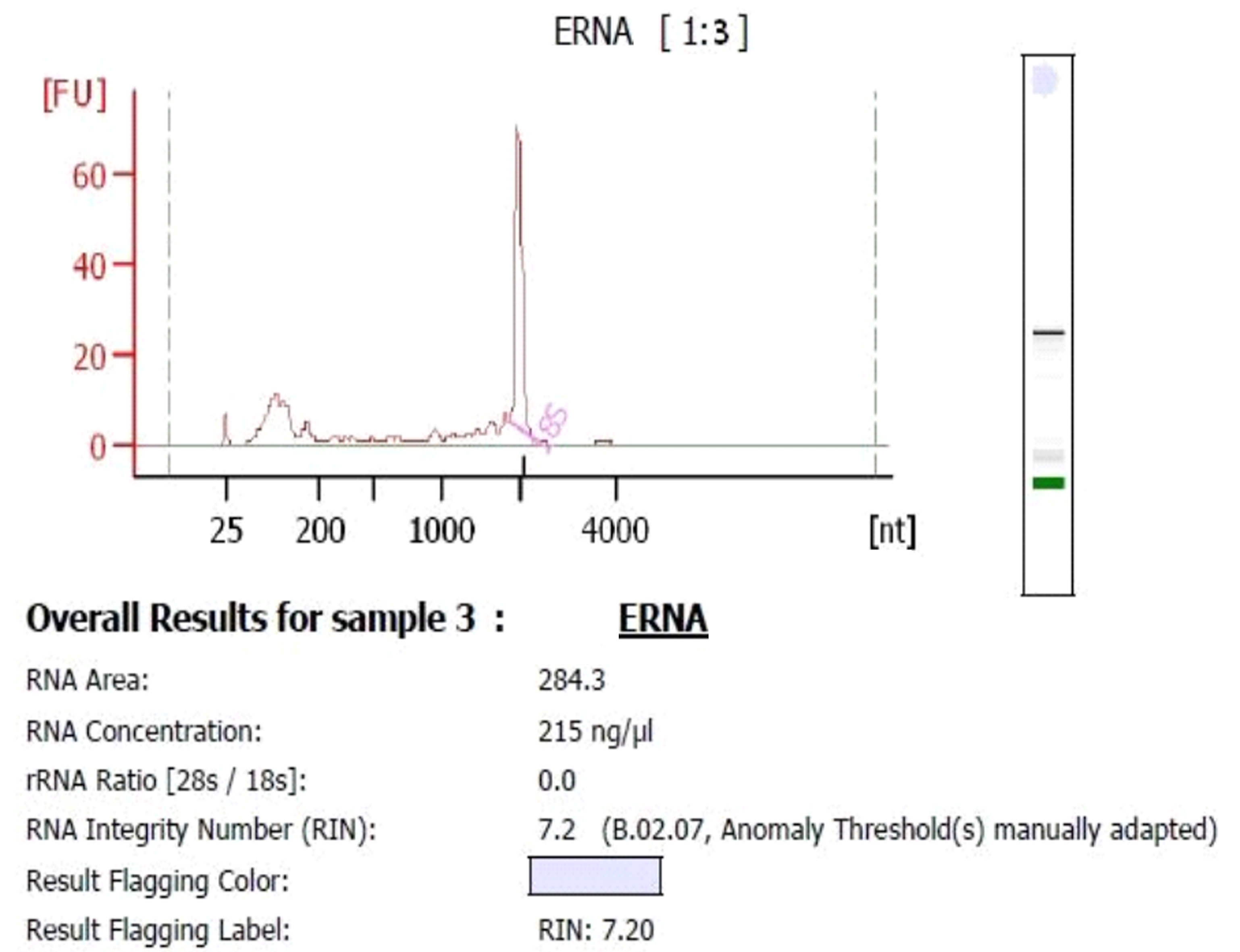

Supplement: Figure S3 — Agilent 2100 Bioanalyzer analysis of total RNA sample extracted from the purified eggs. (TIF) [file pone.0064003.s003.tif]
